# Purification and expression of a novel bacteriocin, JUQZ-1, against *Pseudomonas syringae* pv. *Actinidiae* (PSA), secreted by *Brevibacillus laterosporus* Wq-1, isolated from the rhizosphere soil of healthy kiwifruit

**DOI:** 10.3389/fmicb.2024.1477320

**Published:** 2025-01-07

**Authors:** Yang Shuai, Yi Langbo, Yang Yi, Chen Danni, Peng Qingzhong

**Affiliations:** College of Biology Resources and Environmental Sciences, Jishou University, Jishou, China

**Keywords:** kiwifruit canker, rhizosphere bacterium, *Brevibacillus laterosporus*, bacteriocin, biological control

## Abstract

Kiwifruit canker, caused by *Pseudomonas syringae* pv. *actinidiae* (PSA), has led to significant losses in the kiwifruit industry each year. Due to the drug resistance feature of PSA, biological control is currently the most promising method. Developing biocontrol bacteria against PSA could help solve the issue of drug resistance generated during the chemical control of PSA to a certain extent. In this research, a Wq-1 strain that demonstrated excellent inhibitory activity against PSA was isolated from the rhizosphere soil of healthy kiwifruit. Based on the morphological characteristics and phylogenetic analysis of the 16S rRNA gene sequence, the isolated strain was identified as *Brevibacillus laterosporus* Wq-1. Bacteriostatic proteins were isolated from the cell-free culture filtrate of strain Wq-1 and were found to have a molecular weight of approximately 12 kDa, as determined by sodium dodecyl sulfate-polyacrylamide gel electrophoresis (SDS-PAGE). Liquid chromatography–tandem mass spectrometry (LC–MS/MS) detection revealed that there were several peptides in the target band that were consistent with protein 01021 in the genome. The gene of the 01021 protein was cloned into the plasmid pPICZa, and the recombinant bacteriocin was successfully expressed using the *Pichia pastoris* X33 expression system. The recombinant protein 01021 effectively inhibited the growth of PSA. This is the first report of the protein’s antimicrobial activity, distinguishing it from previously identified bacteriocins. Therefore, we named this bacteriocin JUQZ-1. In addition, our results showed that the protein JUQZ-1 not only exhibited a broad bacteriostatic spectrum but also high thermal and pH stability suitable for harsh environmental conditions., JUQZ-1, a protein with antimicrobial properties and strong environmental tolerance, may serve as a promising alternative to antibiotics.

## 1 Introduction

Kiwifruit is a high-value agricultural crop (Satpal et al., 2021). However, it faces a serious threat from kiwifruit canker worldwide, a disease caused by *Pseudomonas syringae* pv. *actinidiae* (PSA; Scortichini et al., 2012). PSA can infect various organs of the kiwifruit and cause distinctive symptoms, such as ulcers or necrotic spots on the infected areas, some of which release mucus-like substances. These symptoms can lead to blight, garden damage, and other consequences (Renzi et al., 2012). The disease in kiwifruit leads to a decline in fruit quality, which significantly affects the commercial value and market competitiveness of the fruit.

Currently, chemical control is the most important control measure to mitigate kiwifruit canker (Monchiero et al., 2015). Copper compounds (such as copper hydroxide and copper sulfate) and antibiotics (such as streptomycin) are the main control drugs for kiwifruit canker (Cameron and Sarojini, 2014). Some studies have shown that the issue of drug resistance in PSA strains has become a growing concern over time. PSA strains collected from 1984 to 1987 only contained copper resistance genes *copA* and *copB*. However, with the continued use of high copper preparations, two new copper resistance genes, *copR* and *copS*, have been identified in PSA strains (NAKAJIMA et al., 2002). Moreover, a study of PSA strains collected in 2015–2016 revealed that 25% of the strains were already resistant to copper (Colombi et al., 2017). In areas where streptomycin had been used, streptomycin resistance genes were also identified in some PSA strains isolated from Korea and Japan (Han et al., 2004; Vanneste et al., 2008). In addition, the continued use of chemical agents can have adverse effects on the ecological environment, food safety, and human and animal health. Therefore, to ensure the sustainable production of the kiwifruit industry, it is necessary to implement measures for the prevention and control of the causative agent of kiwifruit canker and to explore new control strategies for the protection and development of the kiwifruit industry.

In recent years, several microorganisms with biological control capabilities for inhibiting PSA have been reported. These microbes include *Pseudomonas* spp. (Ali et al., 2022), *Streptomyces* spp. (Kim et al., 2019), *Bacillus* spp. (Wang et al., 2023), *Bipolaris* spp. (Yu et al., 2022), *Aureobasidium* spp. (de Jong et al., 2019), and *Fusarium* spp. (Ma et al., 2023). Furthermore, some studies have indicated that a higher abundance of antagonistic bacteria in the rhizosphere soil of surviving pathogens correlates with reduced plant morbidity, enabling host plants to utilize rhizosphere microorganisms for defense against pathogen infections(Mendes et al., 2011; Berendsen et al., 2012). In a previous study, we performed metagenomic sequencing of kiwifruit rhizosphere soil samples from both healthy and canker-infected kiwifruits and discovered that the healthy samples contained a significantly higher abundance of probiotic bacteria compared to the diseased samples, such as *Bacillus* spp. and *Streptomyces* spp., which are known to inhibit pathogens, fix nitrogen, solubilize phosphorus, and produce phytohormones. These probiotics have potential biocontrol effects (Yang et al., 2023). In summary, we can hypothesize that microbial communities with biocontrol potentiality may be present in the rhizosphere soils of healthy kiwifruit, which can assist the fruit to defend itself against PSA. Metabolites produced by biocontrol bacteria, such as bacteriocins, antibiotics, and various organic compounds, have been widely used in medicine and agriculture due to their biological activities (Ahmad et al., 2017; Ren et al., 2023). These bacteriostatic substances are the main mechanism of action for biocontrol bacteria, especially various bacteriocins and antimicrobial peptides, which do not easily cause bacterial resistance due to their strong inhibitory effects on a wide range of pathogenic microorganisms and their unique antibacterial mechanisms. As a result, they are considered one of the promising candidates to replace traditional antibiotics (Zhang et al., 2014). In addition, as proteins with active functions, bacteriocins and antimicrobial peptides can be degraded by the environment after performing their roles, without contributing to environmental pollution and pesticide residues. They are an excellent option for the prevention and treatment of PSA, in line with the concept of sustainable development.

In this study, we isolated a strain from the rhizosphere soil of healthy kiwifruit that effectively inhibited PSA. The antimicrobial substances were identified and characterized through whole genome sequencing and liquid chromatography–tandem mass spectrometry (LC–MS/MS). Furthermore, they were expressed heterogeneously using a eukaryotic expression system.

## 2 Materials and methods

### 2.1 Strains and samples

*Pseudomonas syringae* pv. *actinidiae* was previously isolated by our research group from the stems of a kiwifruit plant affected by kiwifruit canker and was used as the indicator strain for this experiment. The PSA strain was preserved in the Luria-Bertani (LB) liquid medium containing 20% glycerol at −80°C. *Pichia pastoris* X33 was acquired from Sangon Biotech (Shanghai).

Indicator strains included the following: *Proteusbacillus vulgaris* ATCC-6896, *Escherichia coli* ATCC-35150, *Burkholderia cepacia* CGMCC-27262, *Staphylococcus aureus* CMCC-26003, *Streptococcus agalactiae* ATCC-12403, *Bacillus subtilis* ATCC-6896, *Bacillus safensis* CGMCC-27263, *Aspergillus niger* ATCC-16404, and *Candida albicans* CMCC-98001. All strains were purchased from Baiou Bowei Biotechnology Co., Ltd. (Beijing).

Rhizosphere soil samples from healthy kiwifruit were collected from Songbai town, Yongshun County, Xiangxi Tujia and Miao Autonomous Prefecture, Hunan Province, at coordinates N28°54′, E110°4′.

### 2.2 Isolation of rhizobacteria

Rhizobacteria were isolated using the plate count method in the Luria-Bertani (LB) medium (Zhou et al., 2012; Huang et al., 2013). We crushed the soil samples into granules, mixed them evenly, and weighed 1 g of the soil samples into conical flasks containing sterile water for dilutions of 10^−3^ times, 10^−4^ times, and 10^−5^ times. The samples were shaken uniformly at 180 r/min for 3 min at room temperature, and then, 50 μL of the mixtures was spread onto the LB medium. After cultivating for 7 days at 28°C, different morphological characteristics of the bacterial strains were randomly selected and purified through repeated streaking onto fresh LB plates. The purified colonies were preserved in the LB liquid medium containing 20% glycerol at −80°C.

### 2.3 Screening of the anti-PSA strains

Preparation of PSA-containing plates (Chen et al., 2022): PSA was cultured overnight in the LB medium at 22°C. Then, 100 mL of fresh PSA inoculum was added to 500 mL of the LB medium cooled to 55°C, mixed quickly, and poured onto the plates to create PSA-containing plates. The isolated rhizobacteria were placed onto the PSA-containing plates. The medium was incubated at 22°C until clear zones of inhibited PSA growth became visible.

### 2.4 Assay of antimicrobial activity for the cell-free fermentation broth

The following describes the preparation of cell-free fermentation broth (Teixeira et al., 2009): Bacteria against PSA were cultured in 5 mL of LB liquid medium at 28°C, 180 r/min. After adding 100 μL of the inoculum to 50 mL of LB liquid medium, the culture was fermented at 28°C and 180 r/min. Subsequently, the fermentation broth was centrifuged at 10000 r/min for 10 min at 4°C. Finally, the fermentation broth was filtered through a microporous membrane of 0.22 μm to remove the remaining bacterial cells.

The antagonistic activity of the bacterial fermentation supernatants against PSA was evaluated using the Oxford cup method (Li et al., 2021). After placing Oxford cups on the PSA-containing plates, 200 μL of the fermentation supernatant was pipetted into each Oxford cup. The plates were cultured at 22°C, and bacteriostasis activity was observed after 48 h.

### 2.5 Identification of the antagonistic bacteria

The cellular morphology of the rhizobacteria was observed using Gram staining. Meanwhile, the rhizobacteria were cultured in the LB medium at 28°C for 72 h to observe their colony morphologies.

Genomic DNA from the antagonistic rhizobacteria was extracted using the Rapid Bacterial Genomic DNA Isolation Kit, following the manufacturer’s protocol (Huang et al., 2013). The 16S rRNA gene sequence of the antagonistic strains was amplified by the polymerase chain reaction (PCR) using PA (5′-AGAGTTTGATCCTGGCTCAG-3′) and PB (5′-TTAAGGTGATCCAGCC GCA-3′) primers (Zhao et al., 2021). The reaction mixture was as follows: 25 μL of Taq PCR Mix (2×), 0.4 μM of F/R primers, 0.1 μM of bacterial DNA, and sterile water to a final volume of 50 μL. The PCR procedure included pre-denaturation at 95°C for 2.5 min, followed by denaturation at 95°C for 15 s, annealing at 55°C for 30 s, and extension at 72°C for 1 min for a total of 35 cycles. A final extension was performed at 72°C for 10 min. The amplicons were sequenced, and the sequence was obtained and identified using the NCBI identification service.^1^ The 16S rRNA gene sequences of the Wq-1 strain and type strains of closely related strain species were aligned using MEGA 7.0 software. After removing positions containing gaps and missing nucleotides at both ends of the aligned sequences, the final aligned sequences were constructed into a phylogenetic tree using the neighbor-joining (NJ) method (Waghmode et al., 2011).

### 2.6 Isolation and purification of the bacteriostatic proteins

Ammonium sulfate precipitation: After the fermentation broth was refrigerated at 4°C for 30 min, it was filtered through a microporous membrane of 0.22 μm, and the cooled cell-free fermentation broth was placed on a magnetic stirrer. Ammonium sulfate was then slowly added to 5% of the volume of the fermentation broth each time until the precipitation of the proteins was observed.

Ultrafiltration (Forestier et al., 2022): The ultrafiltration centrifuge tube was pretreated following the instructions. A 500 μL protein sample was added to the centrifuge filter and centrifugated at 4°C and 5,000 r/min for 40 min. First, the sample was filtered using a 30 kDa filter, followed by a 10 kDa filter. The original volume was restored with double-distilled water, and the sample was collected. The inhibitory activity of these isolated samples was determined using the Oxford cup method for PSA.

### 2.7 Identification of the antibacterial proteins

The gel was prepared using a 15% SDS-PAGE gel preparation kit, following the manufacturer’s protocol (Babar et al., 2022). The electrode buffer was prepared using the following components: 15.1 g of Tris, 94 g of Glycine, 5 g of SDS, and 5,000 mL of H_2_O. Dye solution preparation included the following: 1.5 g of coomassie brilliant blue R250, 300 mL of carbinol, 60 mL of glacial acetic acid, and 140 mL of H_2_O. The preparation of decolorizing solution included the following: 300 mL of carbinol, 300 mL of glacial acetic acid, and 400 mL of H_2_O. The electrophoretic conditions were set at 120 V for 100 min.

After cutting the target strip, it was rinsed in a large amount of sterile water, placed into a 1 mL centrifuge tube, and stored in sterile water at 4°C. The target strip was then stored in Drikold and sent to the APTBIO company. The samples were analyzed using LC–MS/MS.

The identified amino acid sequence was analyzed using BLAST searches in the NCBI, UniProt, and ADP3 databases (Wang, 2023), and all similar sequences were downloaded. The sequence similarity of the protein with homologous proteins and bacteriocins was assessed using the Clustal W program.

### 2.8 Complete genome sequencing and information analysis

*B. laterosporus* Wq-1 was cultured in the LB liquid medium overnight at 28°C, 180 r/min for 24 h. The fermentation broth was centrifuged at 8,000 r/min for 5 min. The collected cells were sent to Sangon Biotech (Shanghai) for genome sequencing using the PacBio RS II platform. GeneMarkS (Besemer et al., 2001) software was used to analyze the whole genome sequence of Wq-1 and annotate the function elements of the gene and protein (Coudert et al., 2023).

### 2.9 Allogenic expression of the bacteriocin

To facilitate expression, the signal peptide of protein 01021 was excised, and the protein 01021 codon was optimized. The enzyme cutting sites *EcoR I* and *Acc I* were selected, and 6 × his tags were added to the C-end of the protein to facilitate purification. The synthesized gene was linked to pPICZa, and the recombinant plasmid was named PA-01021. The plasmid PA-01021 was then transfected into *E. coli* DH5a to obtain *E. coli* DH5a-PA-01021.

The *E. coli* DH5a-PA-01021 strain was cultured in LB liquid medium (containing 100ug/mL zeocin) overnight at 28°C, 180 r/min for 24 h. The fermentation broth was centrifuged at 8,000 r/min for 5 min, and the cells were collected. The PA-01021 plasmid was extracted from *E. coli* DH5a-PA-01021 using a plasmid extraction kit. The PA-01021 plasmid was linearized by selecting *Pme I*. *P. pastoris* X33 was cultured in 5 mL YPD liquid medium at 28°C, 280 r/min for 24 h. The *P. pastoris* X33 cells were cleaned with sterile water and 1 mol/L of sorbitol solution. Then, 200 μL of the *P. pastoris* cells was mixed with 10 μL of the linearized PA-01021 plasmid, and the mixed solution was then transferred into a 2 mm electroporation cuvette for electroporation transformation.

The electro-transformed *P. pastoris* X33-PA-01021 was spread on the YPD medium containing 100 ug/ml zeocin and was cultured at 28°C for 48 h. A single colony was selected and cultured at 28°C for two generations. Then, the total DNA of *P. pastoris* X33-PA-01021 was extracted using the Rapid Yeast Genomic DNA Isolation Kit, and subsequently we identified it by PCR. The PCR procedure was similar to 2.5. The 01021 gene was amplified by PCR using primers AOX5 (5′-GACTGGTTCCAATTGACAAGC-3′) and AOX3 (5′-GGCAAATGGCATTCTGA CAT-3′), and the PCR products were then sent to Sangon Biotech (Shanghai) for sequencing.

The *P. pastoris* X33 PA-01021 transformant was cultured in 50 mL BMGY medium overnight at 28°C, 280 r/min for 16 h. The fermentation broths were centrifuged for 5 min at 4°C 6000 r/min, and the cells were collected. The cells were resuspended in 50 mL of BMMY medium. The expression was induced by adding methanol with a final concentration of 0.5%, and then every 24 h, methanol with a final concentration of 0.5% was added. *P. pastoris* X33 transferred to blank pPICZa was used as the control group. After 72 h, the fermentation broths were centrifuged for 5 min at 4°C and 10,000 r/min, and the supernatant was collected. SDS-PAGE was used to detect its expression condition, and the inhibitory activity of the supernatant was detected for PSA using the Oxford cup method.

### 2.10 Purification and identification of the recombinant bacteriocin

The *P. pastoris* X33 PA-01021 fermentation supernatant was collected. The extracellular protein was collected through ammonium sulfate precipitation. The protein was then re-dissolved in 0.1 mol of phosphate buffered saline (PBS) solution and desalted for future use. Equilibrium buffer, wash buffer, and elution buffer were configured according to the instructions for Ni-NTA Agarose. The pH of all buffers and the protein sample was adjusted to 8.0. The Ni-NTA Agarose was transferred to 20 mL of a gravity column. The column was flushed with 5-column volumes of ultra-pure water to remove ethanol, and the equilibrium buffer was added to balance the Ni-NTA Agarose. The protein samples were then added and incubated at room temperature for 2 h, and the gravity column valve was opened to allow the solution to flow out. Then, the wash buffer with 5-column volumes was used to eluate the impurity protein. Finally, the elution buffer was used to eluate the target protein, and the eluent and outflow liquid were collected. The purification effect was identified using SDS-PAGE. The purified sample strips were sent to Sangon Biotech (Shanghai) for LC–MS/MS identification.

The purified sample strips were rinsed and decolorized. The sample strips were rinsed with NH_4_HCO_3_ and C_2_H_3_N to dehydrate them. DTT was added to the samples for reduction, and an IAA solution was added for alkylation. Then, the strips were washed. Finally, the sample strips were dehydrated. Trypsin was added to the sample, followed by the addition of NH_4_HCO_3_ (containing 10% ACN). The mixture was then digested overnight. Once enzymolysis was complete, extraction liquid (67% C_2_H_3_N, 2% CH_2_O_2_) was added, followed by ultrasonic treatment, centrifugation, concentration, and drying. Sample processing was then completed.

The samples were redissolved in Nano-LC mobile phase A (0.1% CH_2_O_2_) for online LC–MS analysis. The samples were loaded onto a nanoViper C18 pre-column (3 μM, 100 Å) and then rinsed for desalting. The liquid phase was provided by Easy-nLC 1,200 nL Liquid Phase System (ThermoFisher, United States). The samples were then separated using an analytical column (C18 reverse-phase chromatographic column, 75 μM × 25 cm, C18-2 μM, 100 Å) after desalting on a pre-column. The gradient used in the experiment involved increasing mobile phase B (80% C_2_H_3_N, 0.1% CH_2_O_2_) from 5 to 38% within 30 min. Mass spectrometry was performed using the Thermo Fisher Q Exactive system, combined with the nanometric spray Nano Flex ion source (ThermoFisher, United States). The spray voltage was 1.9 kV, and the heating temperature of the ion transport tube was 275°C. The scanning mode of the mass spectrometry was data-dependent analysis, with a primary MS resolution of 70,000, a scanning range of 350-2000 m/z, and a maximum injection time of 100 ms. Up to 20 secondary spectra with charges ranging from 2+ to 5+ were acquired during each DDA cycle, with a maximum injection time of 50 ms for secondary mass spectrometry ions. The collision chamber energy (high-energy crash-induced dissociation, HCD) was set to 28 eV for all precursor ions, and the dynamic exclusion was set to 25 s.

The WIFF file of the MS was converted into an MGF format file using ProteoWizard (3.0.10577 64-bit), and the MGF file was imported into Mascot (V2.3.02) for protein identification. The search parameters were as follows: the database was UniProt, the enzyme was trypsin, and the maximum allowable missing cutting site was 1. The fixed modification included carbamidomethylation (C); the variable modifications included acetylation (Protein N-term), deamidation (NQ), Gln- > pyro-Glu (N-term Q), Glu- > pyro-Glu (N-term E), and oxidation (M). The MS tolerance was 20 ppm, and the MS/MS tolerance was 0.05 Da. Identification was considered successful when the protein score C.I. % was greater than 95%.

### 2.11 Sensitivity of the bacteriocin to temperature, pH, and UV

To determine the stability of the bacteriostatic proteins exposed to ultraviolet light, the samples were treated using a UV lamp for 1 h, 3 h, 5 h, 8 h, and 11 h. The remaining antibacterial activity of the samples was determined using the Oxford cup method. The indicator was PSA, while the control was a sample without UV treatment.

The bacteriostatic proteins were kept at 100°C for 1 h, 121°C for 30 min, and 25°C for 15 days, respectively. Among them, the proteins that were kept at 100°C were treated in a water bath, and the proteins that were kept at 121°C were treated in a sterilizer (Naimah et al., 2018). After the treatment, the samples were cooled to room temperature, and the residual antibacterial activity was assessed using the Oxford cup method. The used indicator was PSA. An unheated protein sample was used as the control.

To determine the pH stability of the bacteriostatic proteins, the pH buffer of the purified samples was adjusted to a range from 2 to 11 (in increments of 2 pH) using 4 M NaOH or 4 M HCl (Qi et al., 2021). The samples were then incubated at 25°C for 4 h, after which the pH of each sample was readjusted to 7.0. The remaining antibacterial activity of the samples was determined using the Oxford cup method. The used indicator was PSA, and the control was a sample without pH treatment.

To determine the antimicrobial spectrum of the bacteriostatic proteins, the antibacterial activity of the purified samples was determined using the Oxford cup method. The control was double-distilled water.

## 3 Results

### 3.1 Isolation and identification of the antagonistic strain

The soil samples were gradually diluted and then evenly spread onto LB medium. After 7 days, a substantial number of microbial colonies appeared. Among the different dilution gradients of the soil samples, the 10^−4^ gradient exhibited the most effective separation, with evenly distributed microbial colonies and optimal gaps between them. A total of 93 strains of diverse microorganisms were isolated from the rhizosphere soil samples of kiwifruit, including 30 strains of fungi, seven strains of actinomycetes, and 56 strains of bacteria. After 63 bacteria were co-cultured separately on PSA-containing medium at 22°C for 3 days, it was found that four bacteria demonstrated significant inhibitory activity against PSA. The diameter of their inhibition zones against PSA ranged from 9 to 19 mm. The growth cycle on the LB medium varied from 3 to 7 days. No evident inhibitory activity was observed in the remaining strains of the isolates.

**Figure 1:**
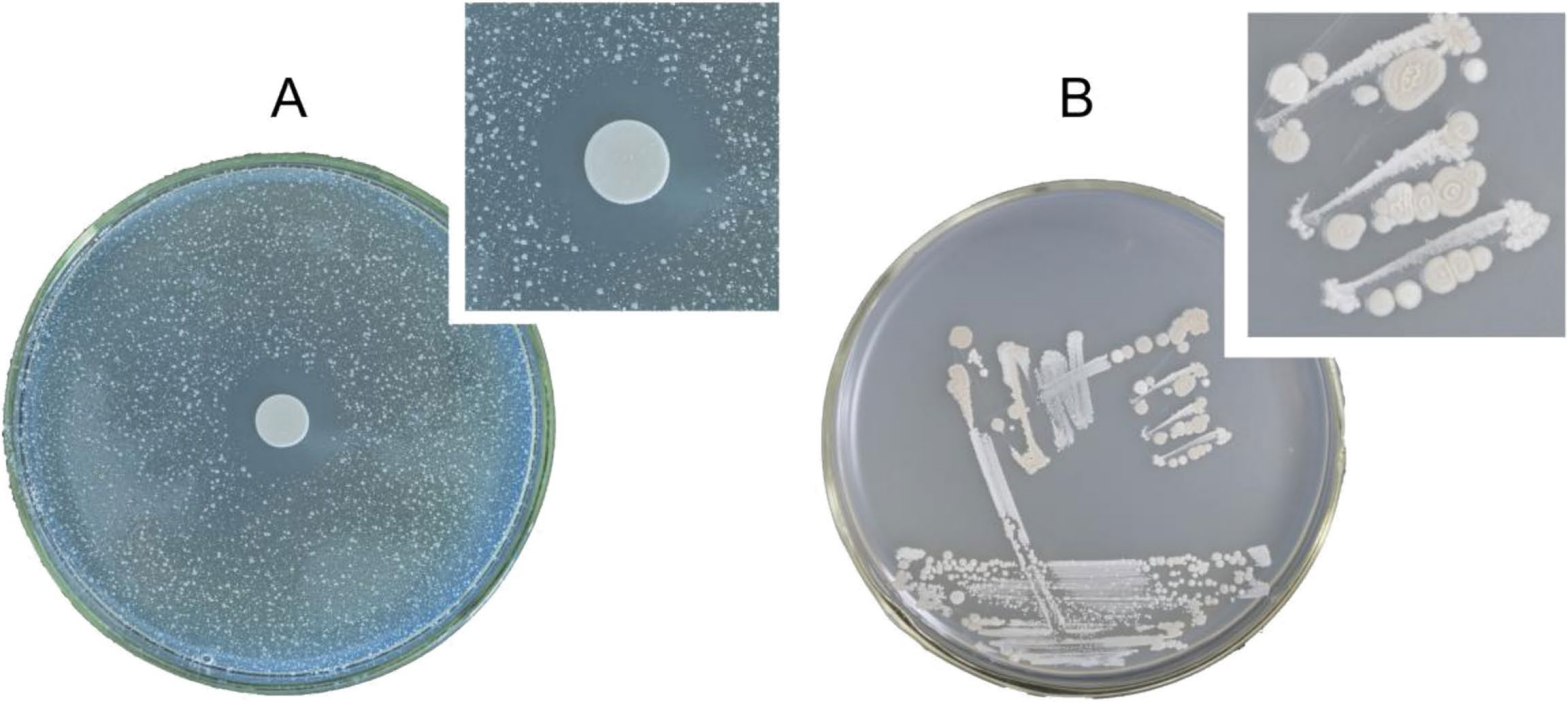
(A) Inhibition effect of strain Wq-1 on PSA. (B) Morphological characteristics of strain Wq-1 were cultivated in LB medium at 28°C for 72 h.

The Oxford Cup method was used to assess the PSA-inhibiting activity of the cell-free fermentation broths derived from the four bacteria. The results revealed that the cell-free fermentation broths of three bacteria exhibited significant inhibitory effects on PSA. The inhibition zone diameter of these three bacteria against PSA ranged from 9 to 15 mm. Notably, the bacterial cell and cell-free fermentation broth of one white bacterium displayed the highest antagonistic activity, with a 15 mm inhibition zone diameter (Figure 1A). Consequently, the white bacterium was selected for further investigation of its bacterial characteristics and antagonistic activity.

**Figure 2:**
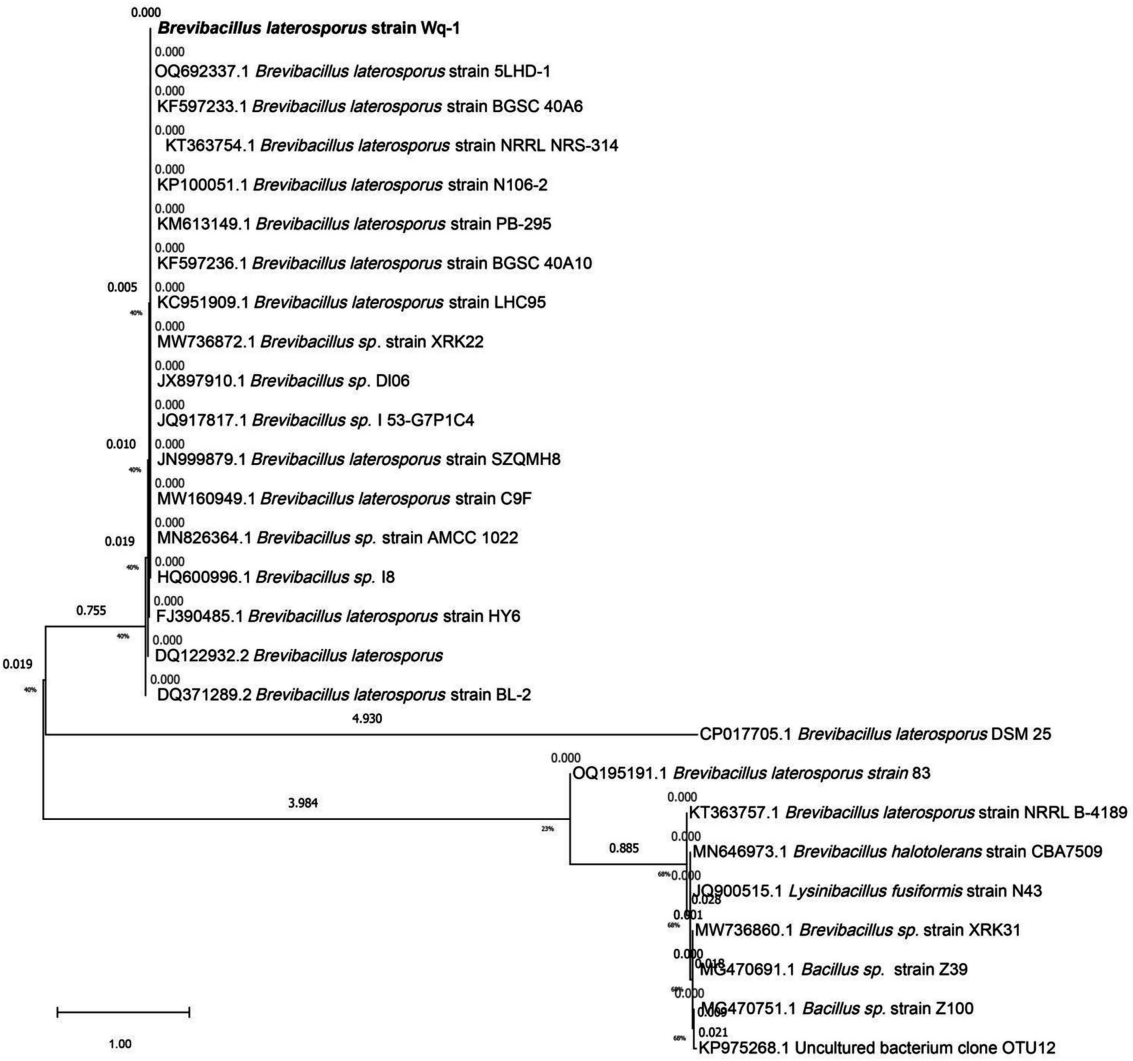
Phylogenetic tree based on the 16S rDNA gene sequences of strain Wq-1. The tree was constructed using the neighbor-joining method in the MEGA program, version 7.0. The bootstrap values were obtained from 1,000 replications.

The white bacterium was cultivated in the LB medium at 28°C for 72 h. Its colony morphology was characterized by a milky-white appearance with concentric circles, as well as surface drying and folding (Figure 1B). A fresh colony was taken for Gram staining, and under a microscope, it was observed that the cells were short and rod-shaped, and the Gram stain was positive. The PCR amplification product size of the Wq-1 strain was 1,360 bp (NCBI accession: OR618324.1), and 16S rDNA sequences analysis showed a maximum identity of 99.46% with *Brevibacillus laterosporus* NECC11213 (PP779784.1). In addition, in the phylogenetic tree (Figure 2), strain Wq-1 clustered with type strain *B. laterosporus* 5LHD-1. Strain Wq-1 was conclusively identified as *B. laterosporus* based on both molecular and morphological characteristics. Therefore, the Wq-1 strain was designated as *B. laterosporus* Wq-1.

### 3.2 Purification of the antimicrobial protein from the Wq-1 strain

Ammonium sulfate was slowly introduced into the cell-free fermentation broth of Wq-1. Protein precipitation was initiated when the ammonium sulfate concentration reached 20%. As the concentration of ammonium sulfate in the cell-free fermentation broth increased, more protein precipitates became evident. Upon reaching 70% ammonium sulfate concentration in the cell-free fermentation broth, the amount of protein precipitates stabilized in the fermentation solution.

The experiment showed that when the ammonium sulfate concentration reached 35%, the protein precipitates harvested through centrifugation exhibited antibacterial activity. However, after collecting the remaining protein precipitates between 35 and 70% ammonium sulfate concentration, the isolated protein no longer retained antibacterial activity. Therefore, the bacteriostatic proteins in the fermentation broth of *B. laterosporus* Wq-1 were all precipitated when the ammonium sulfate concentration reached 35%.

The protein precipitates collected through centrifugation were subsequently re-dissolved in a 0.05 mol/mL PBS solution (pH 7.0). Notably, only approximately 0.02 g of precipitate dissolved per 1 g of precipitate. Then, the soluble portion was separated from the insoluble portion. To prevent interference from salt in the sample during subsequent experiments, the protein sample was desalted using a 3 kDa ultrafiltration centrifuge tube.

The soluble and insoluble fractions of the protein were tested for their bacteriostasis activity against PSA using the Oxford Cup method. The experiment revealed that the inhibitory activity of the insoluble fraction was approximately 20% compared to the dissolved fraction. Therefore, the majority of the bacteriostatic protein precipitates in the cell-free fermentation broth of Wq-1 were re-dissolved in a 0.05 mol/mL PBS solution, with only a small amount of bacteriostatic proteins remaining in the insoluble precipitation. Thus, the soluble precipitated fractions were selected for subsequent purification.

The protein samples were initially placed in a 30 kDa ultrafiltration centrifuge tube for first-stage purification to obtain <30 kDa and > 30 kDa fractions. Bacteriostatic experiments indicated that the inhibitory activity against PSA was concentrated in the <30 kDa fraction. Subsequently, the <30 kDa fraction underwent second-stage separation using a 10 kDa ultrafiltration centrifuge tube to obtain <10 kDa and > 10 kDa fractions. The bacteriostatic experiments revealed that the bacteriostatic activity was concentrated in the >10 kDa fraction (see Supplementary material). In conclusion, the molecular weight of the antibacterial protein in the cell-free fermentation broth of Wq-1 ranged from 10 to 30 kDa.

**Figure 3:**
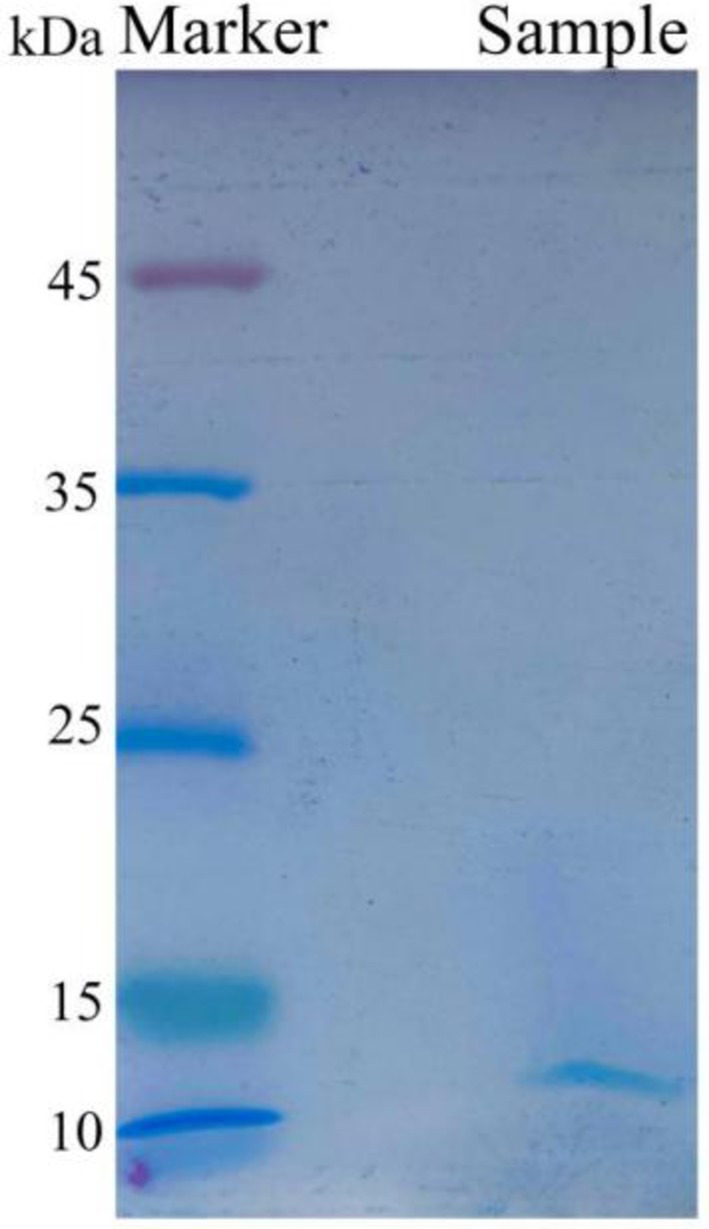
Electrophoresis analysis of the active fraction by SDS-PAGE, isolated from the cell-free fermentation broth of strain Wq-1. A distinct band appeared at 12 kDa. Electrophoresis was performed using a 15% SDS-PAGE gel and a color preparation kit. Electrophoretic condition: 120 V, 100 min.

To precisely determine the molecular weight of the antibacterial protein of strain Wq-1, the ultrafiltered activity fraction was analyzed using SDS-PAGE. The results showed the presence of a single band at 12 kDa in the ultrafiltered activity fraction (Figure 3). In summary, the antimicrobial substances produced by *B. laterosporus* Wq-1 were inhibitory proteins with a molecular weight of 12 kDa.

### 3.3 Identification of the antimicrobial protein from strain Wq-1

**Figure 4:**
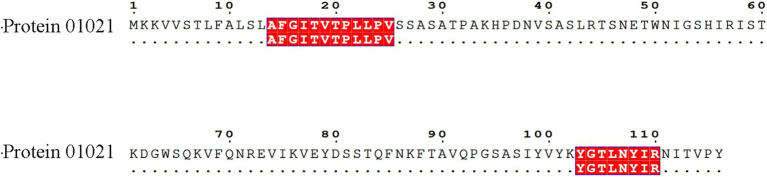
Comparison of the peptide segments identified from the cell-free fermentation broth of strain Wq-1 with the predicted protein sequences from the strain Wq-1 genome.

The 12 kDa protein band is cut into a centrifuge tube, this protein is then enzymatically cleaved and detected by LC–MS/MS. The results showed that two peptide segments had high scores and IBAQ values in the 12 kDa active protein sample. Meanwhile, the whole genome sequence (NCBI accession: CP136163.1) of *B. laterosporus* Wq-1 was analyzed using GeneMarkS software, and a total of 5,025 proteins were predicted. The two peptide segments (AFGITVTPLLPV and YGTLNYIR) identified by LC–MS/MS were compared with the GeneMarkS software output result of the Wq-1 genome sequence (Figure 4), and one protein was obtained, protein ID: PROKKA-01021. Therefore, we concluded that the active substance against PSA in the cell-free fermentation broth of *B. laterosporus* Wq-1 was protein 01021.

**Figure 5:**
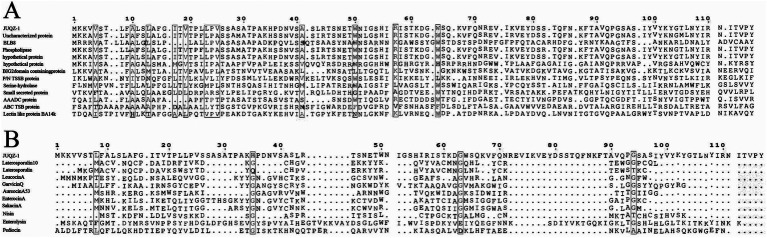
Comparison of the amino acid sequence of protein 01021 with homologous proteins and other bacteriocins. (A) Amino acid sequence comparison between protein 01021 and its congeners. P/N TSSB protein: Peptide/nickel transport system substrate-binding protein; AAADC protein: Amino acid- adenylation domain-containing protein; ABC TSB protein: ABC transporter substrate-binding protein. (B) Amino acid sequence of protein 01021 compared with other bacteriocins.

The sequence of protein 01021 was analyzed using BLAST searches in the NCBI, UniProt, and ADP 3 databases. The homologous sequence was compared with the amino acid sequence of protein 01021. The analysis revealed that protein 01021 is most closely related to an uncharacterized protein (NCBI accession number WP168420414.1), with 100% similarity. The second closest match was BLB8, with 99.14% similarity (Figure 5A). The amino acid sequence of protein 01021 was compared with those of other bacteriocins. The comparison showed that that protein 01021 has very low similarity to Laterosporulin (Similarity 11.8%) and the other seven bacteriocins produced by *B. laterosporus*. The most similar sequence identified was Garvicin Q, with only 19.4% similarity (Figure 5B).

### 3.4 Allogenic expression of the bacteriocin

The primitive length of the coding gene for protein 01021 was 351 bp. After removing the signal peptide and adding the enzyme cutting sites EcoR I and Acc I and 6 × his tags, the gene size was 297 bp. The PA-01021 recombinant plasmid was then constructed. After transferring the plasmid PA-01021 into *P. pastoris* X33, seven transformants were selected. The PCR results showed that all seven transformants successfully incorporated the gene 01021, and they were named Q1, Q2, Q3, Q4, Q5, Q6, and Q7, respectively.

### 3.5 Purification and identification of the recombinant bacteriocin

The bacteriostatic experiments showed that the recombinant protein 01021 expressed by *P. pastoris*-Q1, Q3, and Q5 effectively inhibited PSA, while the control group exhibited no bacteriostatic activity. In summary, protein 01021 was successfully expressed in *P. pastoris* X33 and demonstrated high activity in inhibiting PSA.

The recombinant bacteriocin carried a 6 × his tag, so it was purified using the Ni-NTA resin. SDS-PAGE analysis revealed that, compared to the control group, a clear band appeared at 12 kDa, which was consistent with the theoretical molecular weight of the bacteriocin. The purified recombinant bacteriocin also displayed a clear band at 12 kDa.

LC–MS/MS analysis of the purified protein sample revealed that there were peptide segments in the sample (Table 1). All peptides were matched with JUQZ-1, and the sequence coverage rate was 61.6%. The scores of four peptide segments (TSNETWNIGSHIR, EVIKVEYDSSTQFNK, TAVQPGSASIYVYK, and YGTLNYIR) were greater than 26 (Table 1). The identification result was highly reliable.

**Table 1:** Information and reliability evaluation of the LC–MS/MS identified peptide segment.

Theoretical MW (Da)	Determined MW (Da)	Error (ppm)	Score	Peptide sequence
998.5147	998.5185	−3.85	57	YGTLNYIR
1316.5843	1316.5885	−3.18	59	VEYDSSTQFNK
1513.7209	1513.7273	−4.27	85	TSNETWNIGSHIR
1785.8741	1785.8785	−2.46	97	EVIKVEYDSSTQFNK

### 3.6 Characterization of the antimicrobial protein JUQZ-1

The physical properties of bacteriostatic proteins are key criteria for evaluating their practical value. Some experiments were conducted to characterize the ultraviolet, pH, and temperature tolerance of the purified bacteriostatic proteins.

**Figure 6:**
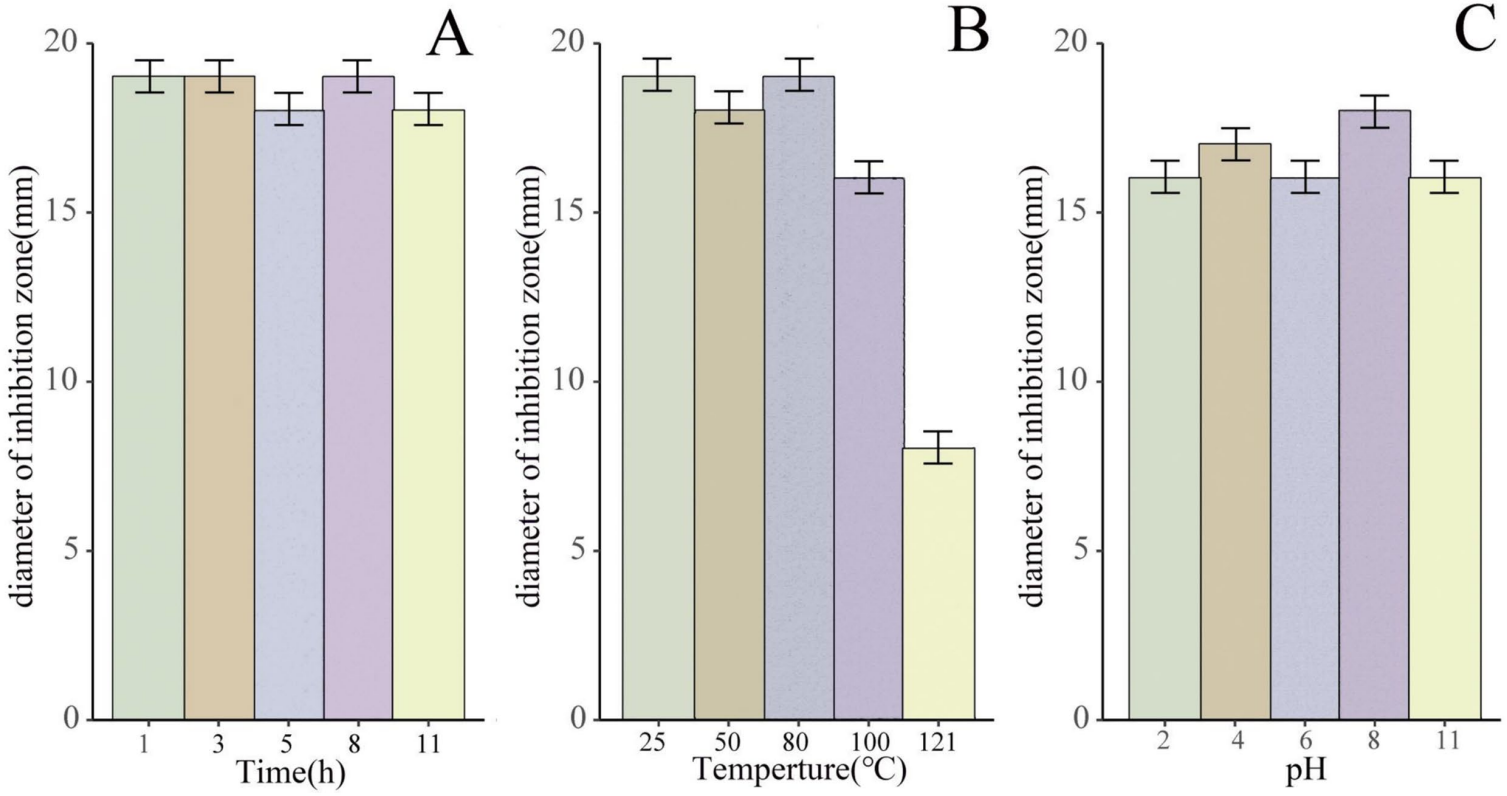
The antimicrobial activity of the bacteriostatic proteins under different pH, temperature, and UV treatment conditions. (A) Bacteriostatic effect of the purified bacteriostatic proteins exposed to UV irradiation for 1 h, 3 h, 5 h, 8 h, and 11 h, using antibacterial size as an indicator. (B) Bacteriostatic effect of the purified bacteriostatic proteins treated at different temperatures. (C) Bacteriostatic effect of the purified bacteriostatic proteins treated at different pH values.

Remarkably, when exposed to UV irradiation for 1 h, 3 h, 5 h, 8 h, and 11 h, these purified bacteriostatic proteins demonstrated sustained antibacterial activity, with no significant decrease in antagonistic activity (Figure 6A). Furthermore, the bacteriostatic activity of the isolated proteins showed no significant decline after 1 h of treatment at 100°C. After a 30 min treatment at 121°C, the bacteriostatic activity remained at 40%, and it maintained 100% activity after 15 days at room temperature (25°C; Figure 6B). The bacteriostatic activity of the antimicrobial protein was preserved at pH 2.0 and pH 11.0 after a 30-min treatment at 25°C. Throughout the pH range from 2.0 to 11.0, the antimicrobial protein showed minimal loss of bacteriostatic activity (Figure 6C).

The antimicrobial spectrum of the protein JUQZ-1 against nine strains is shown in Table 2. JUQZ-1 was found to have broad-spectrum antimicrobial activity against both Gram-positive and Gram-negative bacteria. However, the sensitivity of the Gram-positive bacteria to JUQZ-1 was lower than that of the Gram-negative bacteria to JUQZ-1. Meanwhile, *Aspergillus niger* and *Candida albicans* were completely resistant to JUQZ-1. Overall, JUQZ-1 is a broad-spectrum antimicrobial protein.

**Table 2:** The antimicrobial spectrum of the antimicrobial protein JUQZ-1.

Indicator	Diameter of the inhibition zone (mm)
Gram-negative	
*Pseudomonas syringae* pv. *actinidiae*	18
*proteusbacillus vulgaris*	17
*Escherichia coli*	19
*Burkholderia cepacia*	19
Gram-positive	
*Staphylococcus aureus*	8
*Streptococcus agalactiae*	9
*Bacillus subtilis*	8
*Bacillus safensis*	11
Fungus	
*Aspergillus niger*	0
*Candida albicans*	0

## 4 Discussion

Treating kiwifruit canker, one of the fruit’s primary diseases, continues to attract the attention of scientists. Utilizing the antagonistic relationship between microorganisms to prevent kiwifruit canker has proven to be an efficient strategy among various control techniques (Kim et al., 2019; Ali et al., 2022; Wang et al., 2023). Rhizosphere soil microorganisms have long been in symbiosis with plants, resulting in a profound impact on plant growth. They promote nutrient absorption and provide health protection for plants (Jacoby et al., 2017; Zhou et al., 2021). Therefore, they are potential sources of biocontrol strains. In fact, our research showed that antagonistic strains are present in the rhizosphere soil of kiwifruit, which is consistent with the findings of Yan et al. (2023).

In our experiments, the cell-free fermentation broth from three strains showed good inhibitory activity, indicating that inhibitory activity is mainly caused by extracellular antibacterial components secreted by antagonistic bacteria. According to Saoussen’s research, the production of bacteriostatic substances is the most important mechanism that allows biocontrol bacteria to inhibit plant pathogens (Ben Khedher et al., 2021). Among the strains we screened, strain Wq-1 exhibited the strongest inhibitory effect on PSA. Based on the analysis of the morphological characteristics and 16S rDNA sequence, strain Wq-1 was identified as *Brevibacillus laterosporus* Wq-1. Recent reports have shown that *Brevibacillus laterosporus* can control plant diseases. For example, *B. laterosporus* SN19-1 has been reported to inhibit *Xanthomonas oryzae* pv. *oryzae*, the pathogen responsible for rice bacterial leaf blight (Su et al., 2024). In addition, Li et al. (2021) isolated *B. laterosporus* BL12, which effectively inhibits *Fusarium scabies*, the causative agent of potato common scab. This article is the first to report the antagonistic activity of *B. laterosporus* Wq-1, isolated from kiwifruit rhizosphere soil, against the kiwifruit canker pathogen PSA.

However, several studies have shown that preventing PSA through chemical control is becoming increasingly difficult due to the pathogen’s resistance (Nakajima et al., 2002; Han et al., 2004; Vanneste et al., 2008; Colombi et al., 2017). Using activity-guided fractionation, we determined that the main mechanism by which *B. laterosporus* Wq-1 inhibits PSA is through the production of a 12 kDa bacteriostatic protein. It is difficult for pathogens to resist the attack of antibacterial proteins through common resistance mechanisms (Yan et al., 2021). For example, antimicrobial proteins can penetrate the outer membrane or cell wall of pathogens, bypass the bacterial efflux pump or enzyme system, or directly target multiple sites within the bacteria, making it challenging for pathogens to develop resistance. Therefore, antimicrobial proteins have become a major focus of research in biocontrol and the biopharmaceutical industry (Cui et al., 2021; Yan et al., 2021). To better understand the bacteriostatic protein produced by *B. laterosporus* Wq-1, we sequenced its genome and annotated a large number of proteins. At the same time, the purified samples were analyzed through LC–MS/MS. The significant finding is that the segments from the antimicrobial proteins identified through LC–MS/MS in the extracellular proteins correspond to protein 01021, which is annotated in the Wq-1 genome (Figure 4). This suggested that protein 01021 is a novel bacteriostatic protein that has not yet been fully characterized. We named it JUQZ-1 (NCBI accession: PQ065985).

We successfully determined the coding gene and complete amino acid sequence of JUQZ-1. We found that JUQZ-1 contains a signal peptide, which indicates that JUQZ-1 is different from most bactericins that lack signal peptides. JUQZ-1 may involve more complex processing and modifications during secretion (Ren et al., 2023). Furthermore, it is worth noting that Li et al. (2021) and Wang et al. (2015) used the *E. coli* system to express the proteins BLB8 (NCBI accession: QBP43010.1) and PeBL1 (NCBI accession: AJE60449.1), produced by *B. laterosporus* B8 and *B. laterosporus* A60, respectively, to combat Tobacco mosaic virus (TMV). We observed that the amino acid sequences of the BLB8 and PeBL1 proteins were similar to that of the JUQZ-1 protein identified in this study. This observation is not surprising as it indicates that this type of protein has multiple antibacterial and antiviral functions, a characteristic that is commonly observed (Al Kassaa et al., 2014), such as with staphylococcin 188 (Saeed et al., 2006) and enterocin B (Ankaiah et al., 2018). Although Li et al. (2021) and Wang et al. (2015) successfully expressed the homologous protein of JUQZ-1 using the *E. coli* system, there was no clear evidence of inhibiting pathogenic bacteria. Considering that JUQZ-1 may have a more complex modification process, to preserve its biological activity as much as possible, we selected the *P. pastoris* expression system for recombinant expression. We also selected extracellular expression to restore the modification process of JUQZ-1 in the original host as much as possible (Peng et al., 2012). In addition, this approach facilitated subsequent purification processes. Using *P. pastoris* X33 and pPICZa, we successfully expressed recombinant JUQZ-1, which showed high inhibitory activity against PSA (with an inhibition zone diameter of 22 mm), while no inhibitory activity was observed in the control group. Furthermore, the LC–MS/MS detection results showed that the identified peptides matched with JUQZ-1, which fully proved the inhibitory effect of JUQZ-1 on PSA. This is the first successful heterologous expression of the active bacteriocin JUQZ-1 in *P. pastoris*. *P. pastoris* X33 PA-01021 is expected to be a powerful tool for the industrial production of JUQZ-1.

In this study, it was observed that the bacteriostatic protein JUQZ-1 not only inhibits the kiwifruit canker pathogen PSA but also shows activity against other Gram-positive and Gram-negative bacteria. (Table 1). In addition, JUQZ-1 demonstrated excellent stability to heat and varying pH levels. These characteristics suggest that the bacteriocin JUQZ-1 produced by *B. laterosporus* has significant potential for application in the plant industry.

## Data Availability

The original contributions presented in the study are publicly available. This data can be found here: Brevibacillus laterosporus Wq-1 16S rDNA sequence, GeneBank accession number: OR618324.1 (https://www.ncbi.nlm.nih.gov/nuccore/OR618324.1/). Brevibacillus laterosporus Wq-1 Whole genome sequence, GeneBank accession numbers: CP136163.1 (https://www.ncbi.nlm.nih.gov/nuccore/CP136163.1/). Bacteriocin JUQZ-1 complete CDS sequence, GeneBank accession numbers: PQ065985.1 (https://www.ncbi.nlm.nih.gov/nuccore/PQ065985.1/).

## Footnotes

^1^ http://www.ncbi.nlm.nih.gov
