# Correction: Purification and expression of a novel bacteriocin, JUQZ-1, against *Pseudomonas syringae* pv. *Actinidiae* (PSA), secreted by *Brevibacillus laterosporus* Wq-1, isolated from the rhizosphere soil of healthy kiwifruit

**DOI:** 10.3389/fmicb.2025.1666370

**Published:** 2025-07-28

**Authors:** Frontiers Production Office

**Affiliations:** Frontiers Media SA, Lausanne, Switzerland

**Keywords:** kiwifruit canker, rhizosphere bacterium, *Brevibacillus laterosporus*, bacteriocin, biological control

The funder “Postgraduate Scientific Research Innovation Project of Hunan Province,” “Grant Number CX20231084” to Yang Shuai was erroneously omitted.

The new statement is below:

“The author(s) declare that financial support was received for the research and/or publication of this article. This work was supported by the National Natural Science Foundation of China (grant number 32160009), the Key Research and Development Project of Hunan Province (grant number 2022NK2055), and the Postgraduate Scientific Research Innovation Project of Hunan Province (grant number CX20231084) to Yang Shuai.”

The original version of this article has been updated.

