# Supplementary figures and images for: Purification and expression of a novel bacteriocin, JUQZ-1, against Pseudomonas syringae pv. Actinidiae (PSA), secreted by Brevibacillus laterosporus Wq-1, isolated from the rhizosphere soil of healthy kiwifruit
Source: Front Microbiol. 2025 Jan 7;15:1477320. doi: 10.3389/fmicb.2024.1477320 (PMC11747845; doi:10.3389/fmicb.2024.1477320)

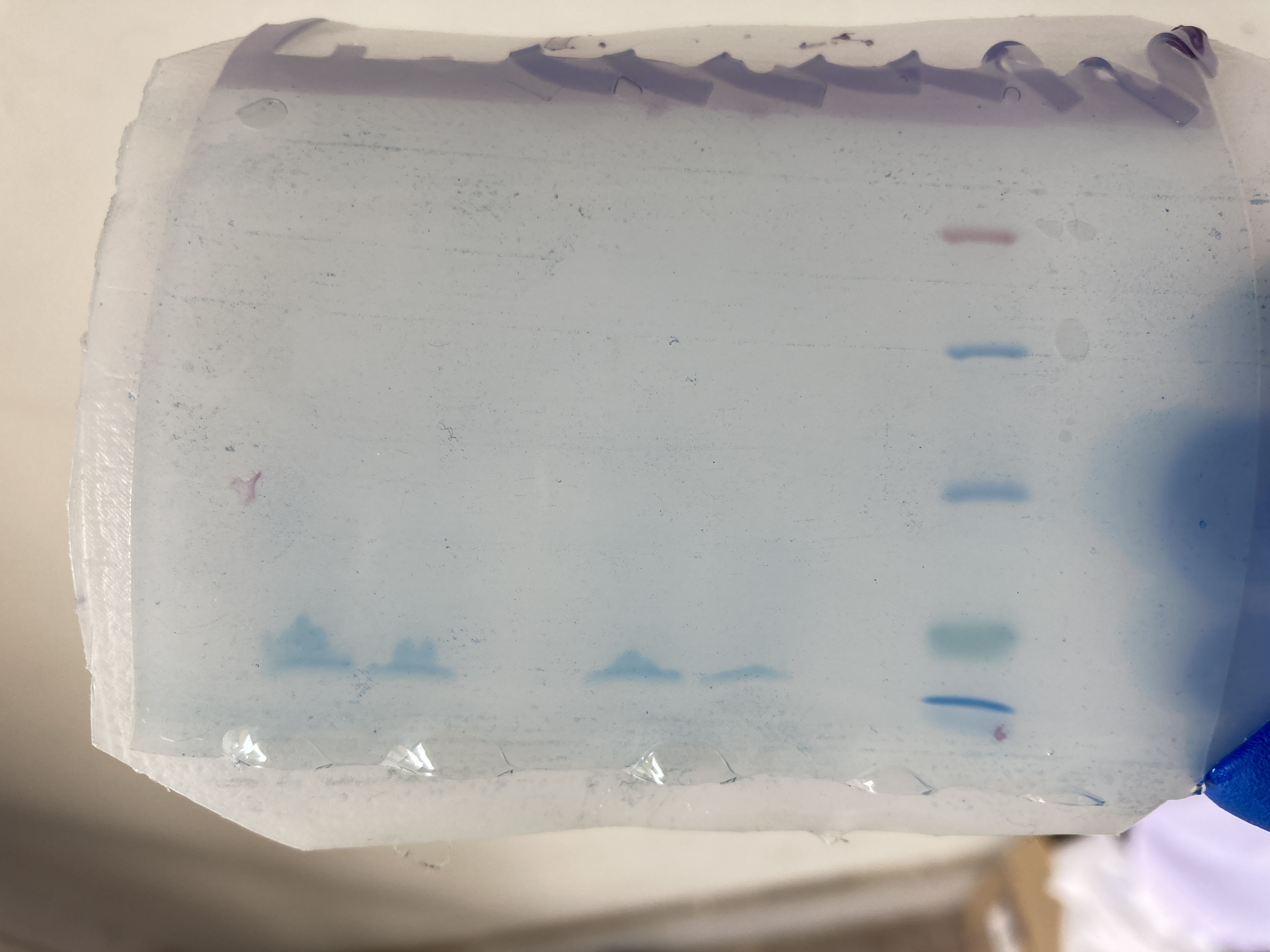

Supplement: Supplementary file 1 [file Image_1.JPEG]

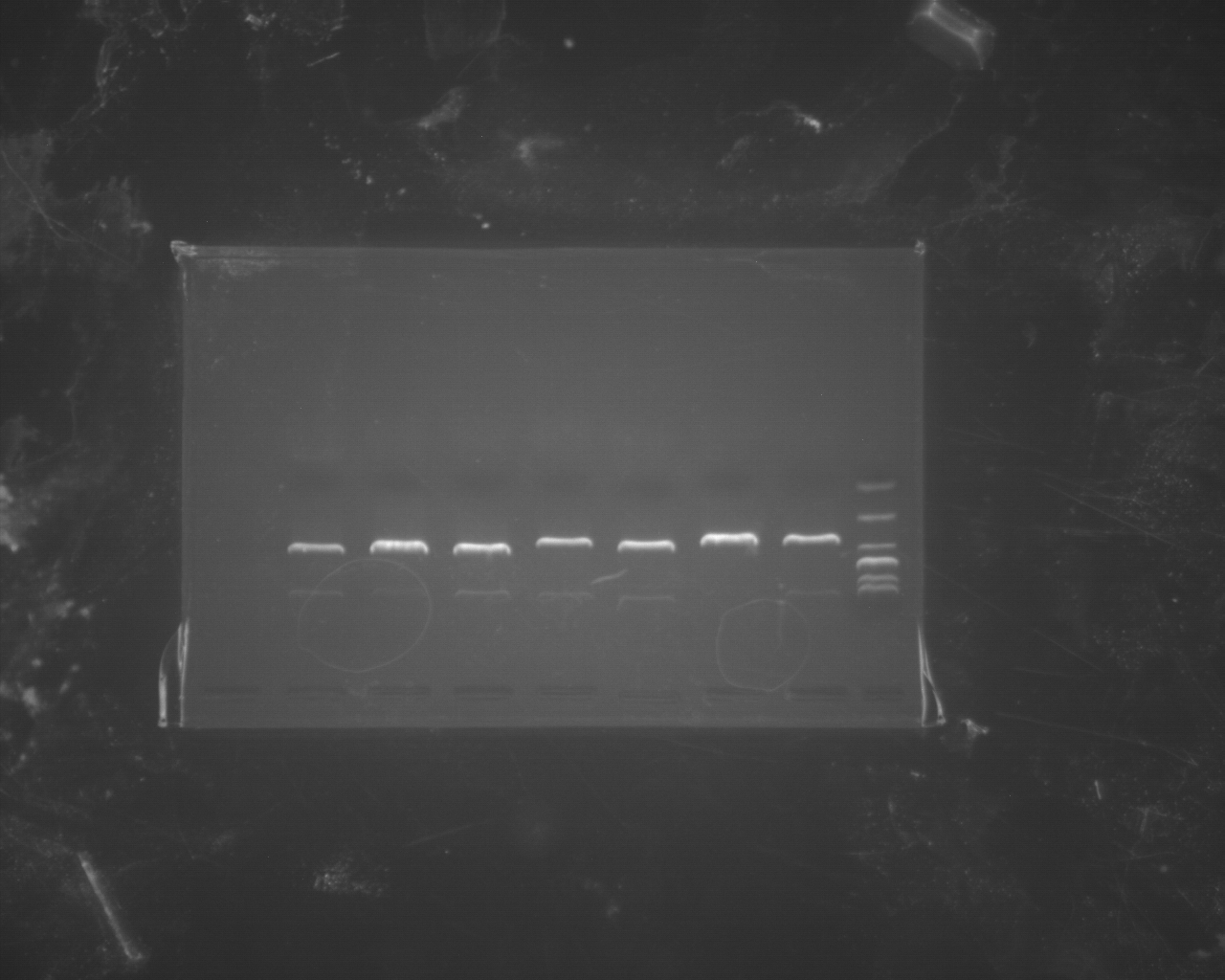

Supplement: Supplementary file 2 [file Image_2.PNG]

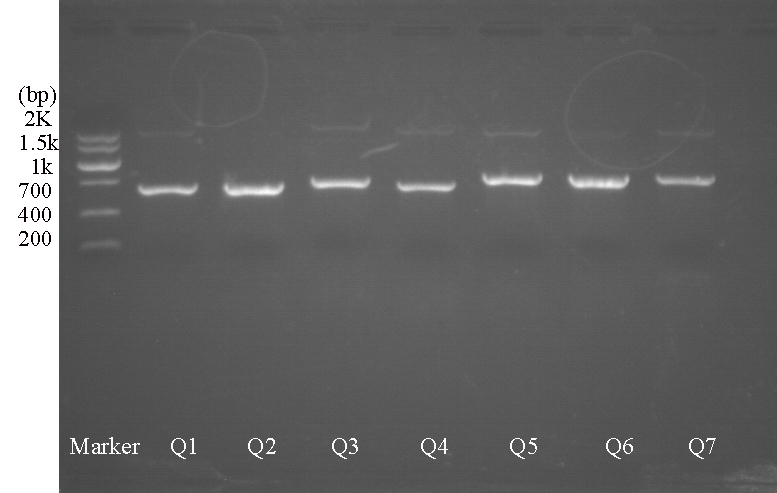

Supplement: Supplementary file 3 [file Image_3.JPEG]

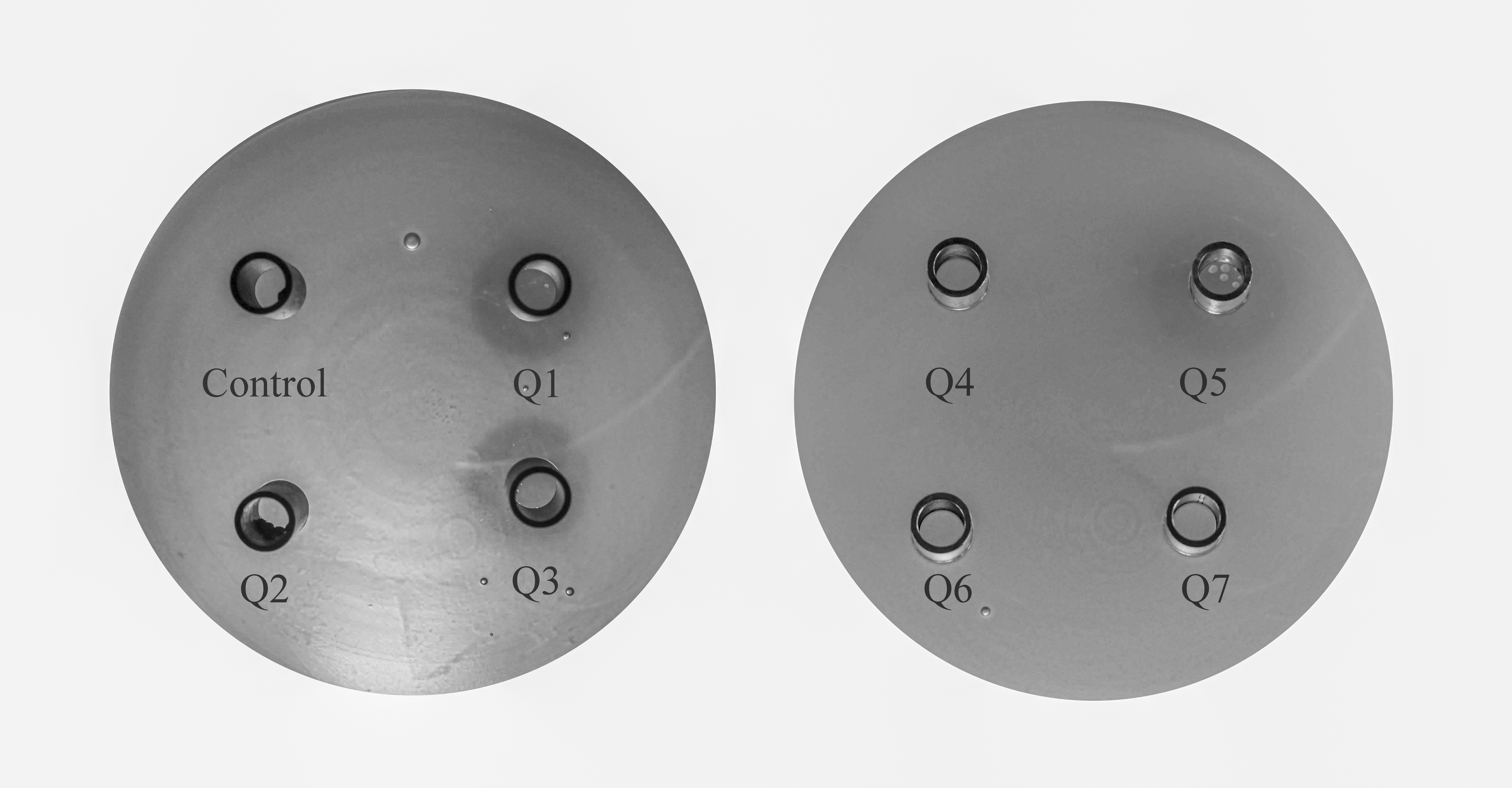

Supplement: Supplementary file 4 [file Image_4.JPEG]

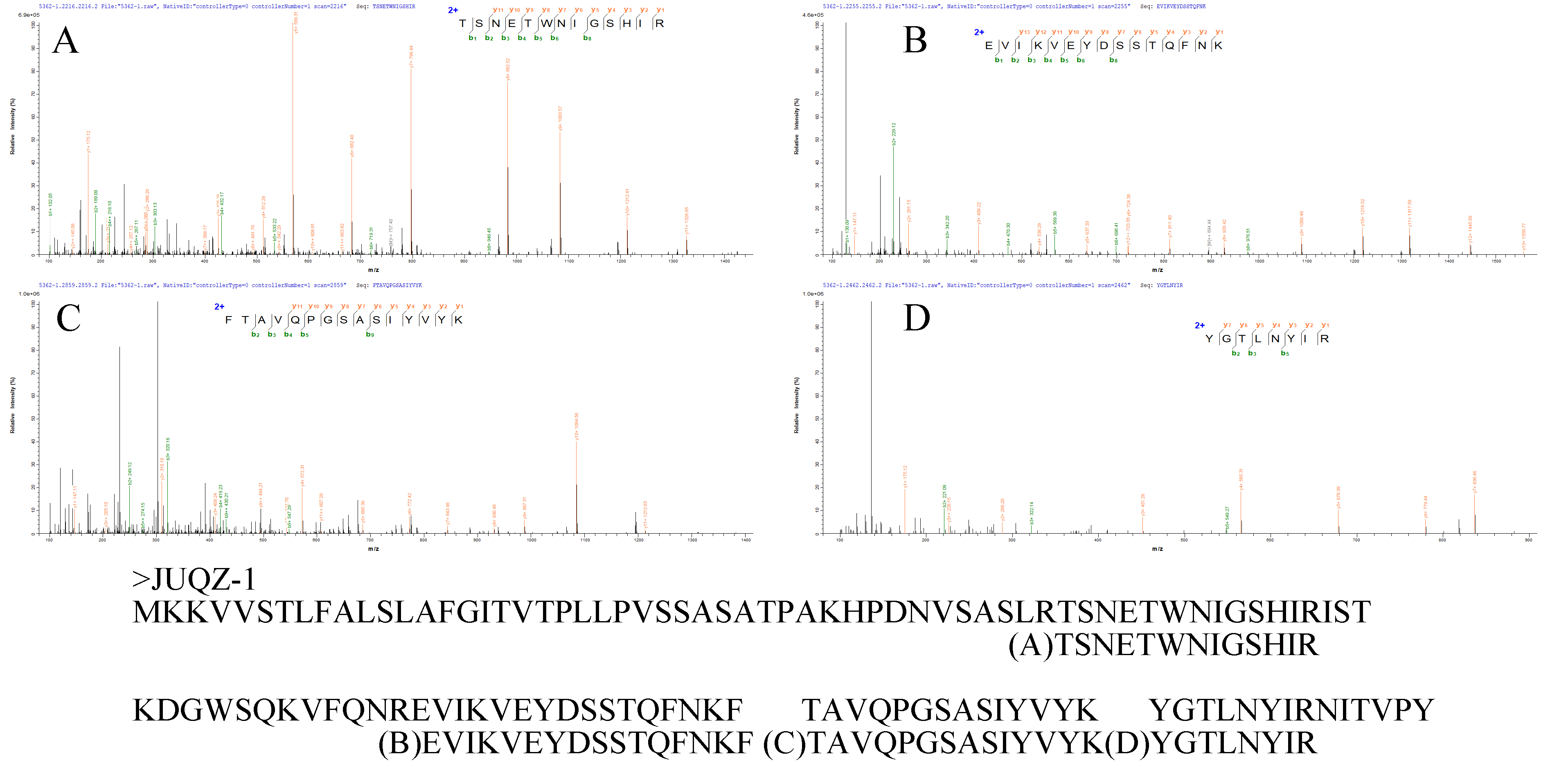

Supplement: Supplementary file 5 [file Image_5.JPEG]

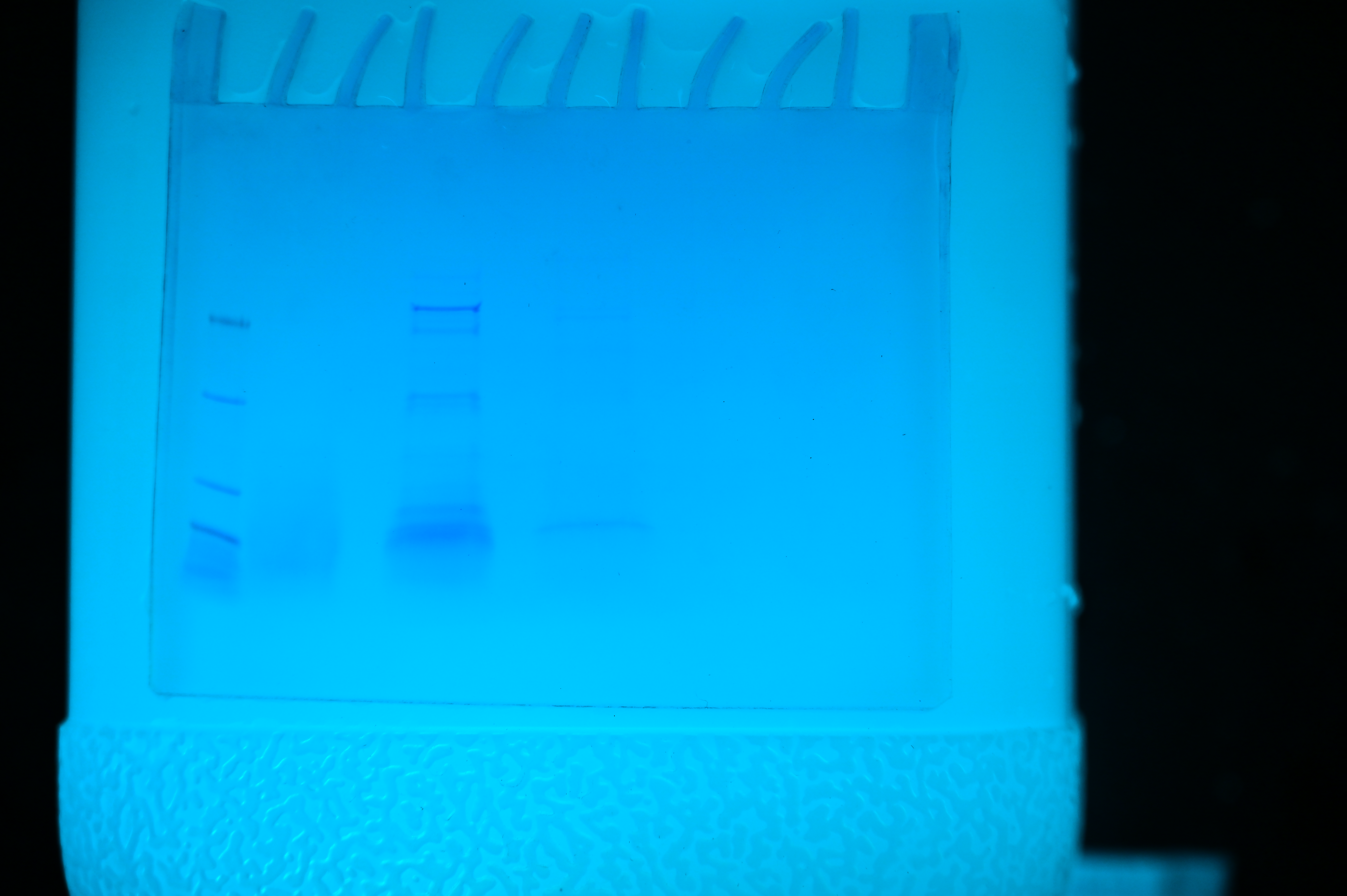

Supplement: Supplementary file 6 [file Image_6.JPEG]

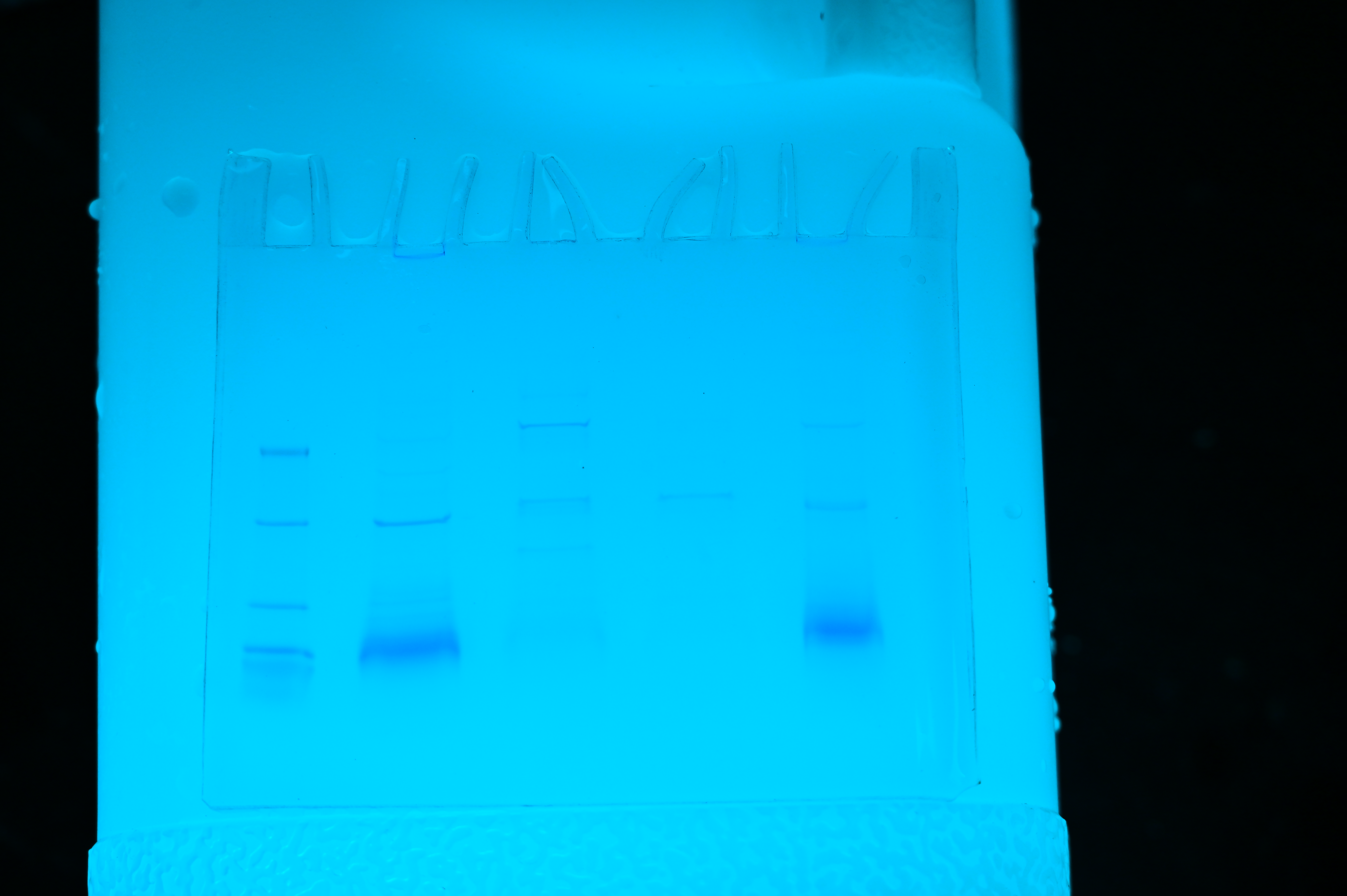

Supplement: Supplementary file 7 [file Image_7.JPEG]

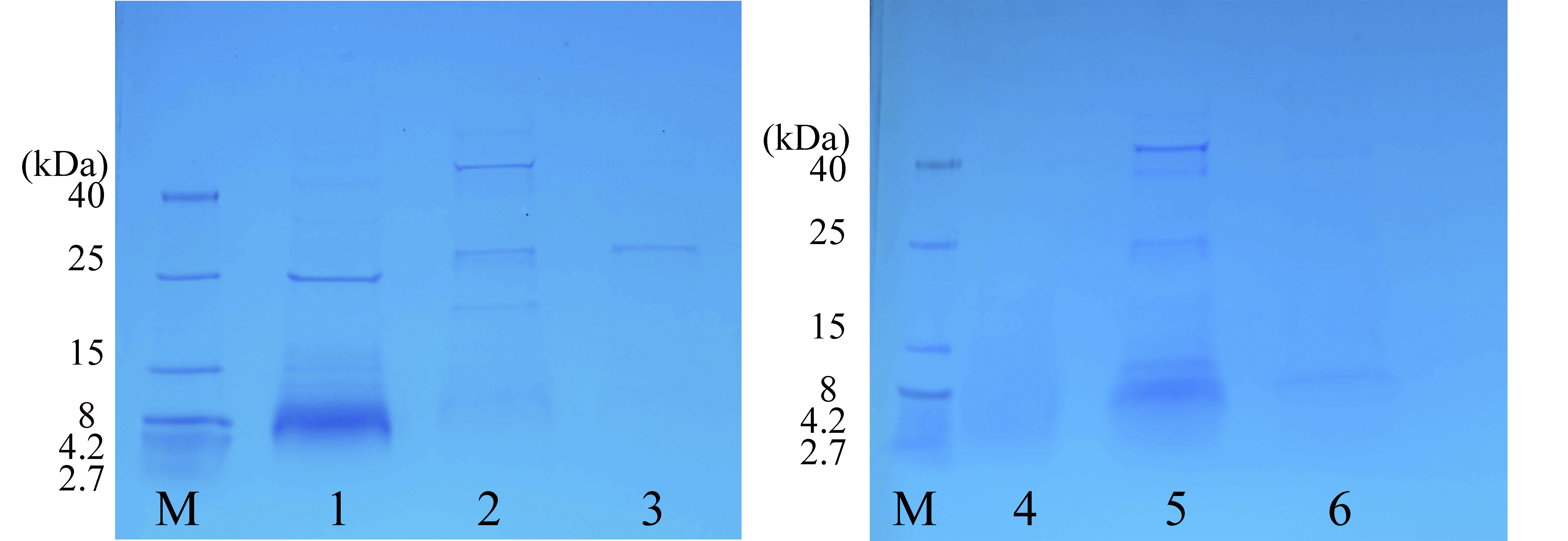

Supplement: Supplementary file 8 [file Image_8.JPEG]

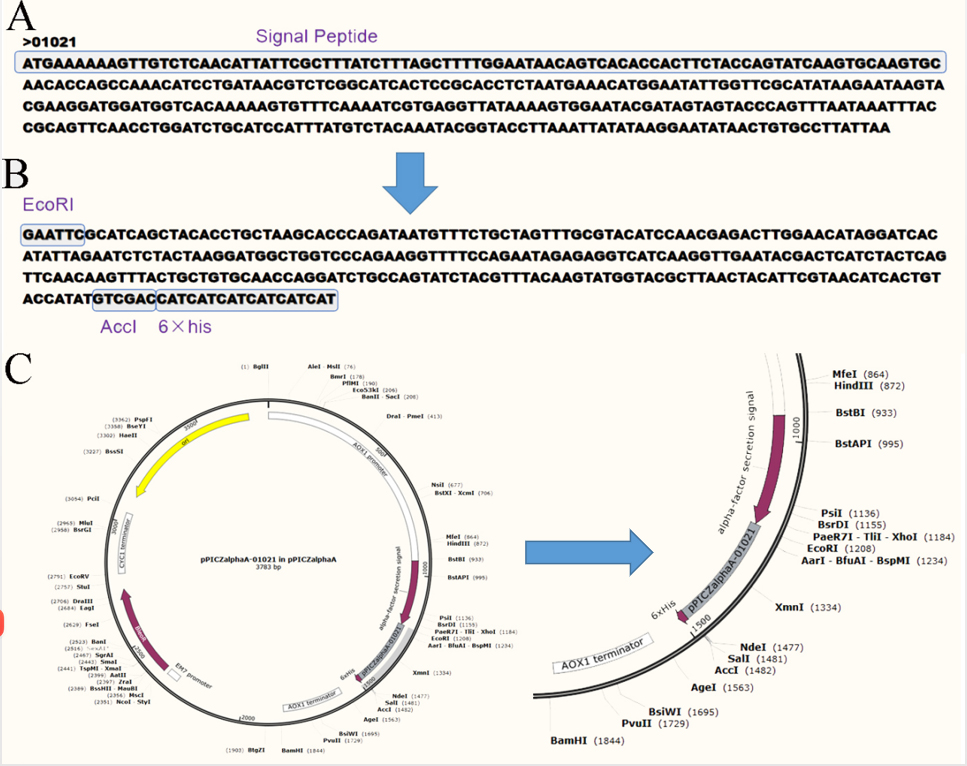

Supplement: Supplementary file 9 [file Image_9.JPEG]
